# Report of the Proceedings of the Chicago Society of Physicians and Surgeons

**Published:** 1873-09-15

**Authors:** 


### Society Reports.

### REPORT OF THE PROCEEDINGS OF THE CHICAGO SOCIETY OF PHYSICIANS AND SURGEONS.

MEETING OF AUGUST 25TH, 1873.

*Plymmar S. Hayes, M.D., Reporter.*

Dr. A. Fisher in the chair. The following named Physicians were elected to membership: Dr. C. W. Burrill, Dr. W. E. Quine, and Dr. B. J. Perry.

The name of Dr. Edwin Powell, of Rush Medical College, was proposed by Dr. J. E. Owens.

On motion of Dr. W. C. Lyman, the following resolution was adopted:

"Invitations to participate in the discussions or transactions of this Society may be extended only to members of other regular medical societies, non-residents, or practitioners connected with regular medical schools or hospitals."

Dr. I. N. Danforth exhibited under the microscope a specimen of "rice-water" discharge, from a person affected with cholera. Dr. Danforth explained the characteristic alterations in the cells of the mucous membrane of the intestinal tract; and Dr. C. J. Simons gave the ante-mortem history of the patient from whom the specimen was obtained.

Dr. J. N. Hyde, the Secretary, read a letter from Dr. A. Poitevin, Vice-Consul of France at Mobile, recommending the use of Ethiop's mineral, in cases of cholera, on the authority of Prof. Cadet, of Rome, Italy. Also, the following report from the Committee on Treatment of Cholera:

REPORT OF COMMITTEE ON TREATMENT.

In consultation with a few of our older physicians who have had most experience in the treatment of cholera during its prevalence in former epidemics, we find a most gratifying uniformity in the views expressed regarding the treatment of this so much dreaded disease.

Without reference to particular theories of pathology, and especial theories of treatment founded thereon, experience seems to have demonstrated that certain remedies are in a measure efficient and successful in arresting the disease, when they can be resorted to in season.

In the active stage of vomiting and purging, small doses, frequently repeated, of calomel and morphine, are recommended by all with whom we have conversed. These remedies, given in form of powder, immediately after each attack of vomiting, will, some part of them, adhere to the coats of the stomach long enough generally to exert their influence. A solution of calomel, acetate of lead, and morphine, thrown well up into the rectum, immediately after a discharge, when the lower bowel is empty, will also be likely to be retained so as to aid materially.

When called in later, after collapse has set in, or in cases that progress on to collapse in spite of treatment, Dr. N. S. Davis places much reliance on the following mixture:

℞.—Acid nitric, ʒ i.

Strychnia, gr. i.

Tinct. opii, ʒ iv.

Syr. simplex, ℥ i.

Aquæ, ℥ iij.

M. ʒ i. every hour, alternating with calomel and morphine powders, as above.

The above mixture is also valuable as a tonic in the convalescence from cholera, when there is nervous prostration and a general relaxed state of the system.

In the painless diarrhœas which so frequently precedes the active onset of the cholera attack, and which seems to result from a generally relaxed condition of the mucous membrane, the following is a favorite mixture with some:

℞.—Arom. sulph. acid ʒ iij.

Sulphate magnesia, ʒ iij.

Tinct. opii. ʒ iij.

Aquæ mentha, ℥ iij.

M. ʒ i. dose, repeated more or less frequently, according to urgency of symptoms. It has the merit of being rather agreeable to the taste, and not likely to offend the stomach.

To the following gentlemen, among others, we are most especially indebted for the above information regarding the treatment of cholera, and all of whom have expressed their confidence in the value of calomel and morphine in the active stage: Prof. H. A. Johnson, Dr. John Bartlett, and Prof. N. S. Davis.

J. H. ETHERIDGE,

H. P. MERRIMAN,

C. G. SMITH,

F. H. DAVIS,

DR. BOYD,

} *Committee.*

Dr. Thos. Bevan, Chairman of the Committee on History of Cholera in Chicago, reported progress, and requested further time for the completion of their report.

The Section on Surgery then presented its Annual Report for 1873.

The Chairman, Dr. W. C. Lyman, read a report on Stricture of the Urethra and Rectum.

Dr. A. S. Peck read a report on the Electro-Therapeutics of Surgery.

Dr. John E. Owens read a report on Staphylloraphy and Uranoplasty, illustrating his remarks by card-board models of the palate and membrane flaps; exhibiting, also, an improved gag.

Dr. John Bartlett described a new and efficient method of producing vaccine vesicles by the aid of fenestrated plaster and cantharidal cerate.

It was announced that two members of the Surgical Section had yet to report.

The Society then adjourned.

